# Editorial: Methods and applications in vascular physiology: 2022

**DOI:** 10.3389/fphys.2023.1264518

**Published:** 2023-08-08

**Authors:** Antonio Colantuoni, Julien V. Brugniaux, Alexey Goltsov, Rosalia Rodriguez-Rodriguez

**Affiliations:** ^1^ University of Naples Federico II, Naples, Italy; ^2^ HP2 Laboratory, INSERM, University Grenoble Alpes, Grenoble, France; ^3^ Institute for Artificial Intelligence, Russian Technological University (MIREA), Moscow, Russia; ^4^ Basic Sciences Department, Faculty of Medicine and Health Sciences, Universitat Internacional de Catalunya, Barcelona, Spain; ^5^ Centro de Investigación Biomédica en Red de Fisiopatología de la Obesidad y la Nutrición (CIBEROBN), Instituto de Salud Carlos III, Madrid, Spain

**Keywords:** vascular physiology, methods, protocols, clinical research, vascular disease, diagnostics

This Research Topic is a part of the Methods and Applications in Physiology series and builds on the successful publication of the “*Methods and Applications in Vascular Physiology: 2022*”. In current issue, we included manuscripts devoted to novel methods and protocols and extension of existing ones covering wide aspects of the vascular physiology at the molecular, cellular, intact tissue, and systems levels. For instance, the articles from Wallis et al. and Schubert et al. present *ex vivo* results describing a novel methodology to assess NO production and discussing the relevance of the different models of myography on isolated blood vessels, respectively. On another hand, *in vivo* studies i) described the interaction between the cardio–ankle vascular index and blood pressure during haemodialysis (Sato et al.), ii) compared the merits of a large array of techniques used to assess aortic systolic and pulse pressure (Bia et al.) and iii) proposed a common outcome measure, namely, the vascular health index (VHI), developed to reconcile multiple measures routinely used to assess vascular function (Menon et al.). A more detailed overview of these manuscripts included as part of this Research Topic is provided below.

The manuscript by Wallis et al. made a substantial contribution to the assessment of NO availability, and therefore vascular function, in intact arteries with fluorescent dyes. Accurate measurement of NO in arteries is problematic since this is an unstable and highly lipophilic free radical. The authors characterised the rhodamine-based DAR-4M AM and copper-based Cu2FL2E fluorescent NO-dyes, and refined their application in rat isolated resistance arteries in a pressure myography or *en face* imaging of endothelial cells. The comparison of both dies revealed that while they similarly responded to NO generated extracellularly from the NO donor SNAP, only DAR-4M responded to endogenous NO production and NO donors within intact arteries. Then, this study proposed a simple and reproducible approach based on the use of the fluorescent rhodamine-based NO dye, DAR-4M AM, for visualising NO production in isolated mesenteric arteries. This novel assay has the potential to be applied to other types of arteries to continuously monitor NO release, comparing basal *versus* stimulated conditions and therefore advance our understanding of vascular function.

Next, Schubert et al. highlighted the importance of studying the mechanisms of regulation of vascular tone by myography (isometric and isobaric) of isolated blood vessels (one of the most physiologically relevant approaches to study the function of cells in the vessel wall), and how they can be complimented by *in vitro* studies on isolated vessels. According to the suggestion of the authors, numerous parameters that are difficult to control *in vivo* can be utilized for a deep understanding of vascular physiology. In this accurate review, the authors reported the design of experiments using myography in combination with methods to measure additional parameters. The list of suggested analyses is very long and interesting; mRNA and protein expression, the protein phosphorylation and signalling pathway activity, endothelium-denuded samples, and membrane potential measurements by calcium fluorimetry. Moreover, the authors illustrated the vascular neuroeffector mechanisms to analyse both the electrical field stimulation of intramural nerves and smooth muscle cells. Finally, the authors proposed to investigate the effects of sympathetic, parasympathetic and sensory nerves and the prejunctional mechanisms of vascular neurotransmission.

Sato et al. were interested to study blood pressure (BP) changes during haemodialysis (HD), utilizing the cardio–ankle vascular index (CAVI) to clarify the role of CAVI in regulating the BP system during HD. They studied 10 patients undergoing 4-h HD (total 57 HD sessions). Changes in the CAVI and various hemodynamic parameters were evaluated during each session. During HD, BP decreased and CAVI significantly increased. Changes in CAVI from 0 to 240 min were significantly correlated with water removal rate (WRR). Changes in CAVI at each measurement point were negatively correlated with ΔBP. Arterial stiffness monitored by CAVI generally increased during HD in nine patients. CAVI elevation was associated with decreased WWR and BP. The authors suggested that an increase in CAVI during HD may reflect the contraction of smooth muscle cells and play an important role in BP maintenance. Therefore, measuring CAVI during HD may be useful to clarify the mechanisms of BP changes.

Bia et al. evaluated the agreement between the non-invasive and invasive estimation of aortic systolic (aoSBP) and pulse pressure (aoPP). They utilized different recording techniques (tonometry, oscilometry/plethysmography and ultrasound), different recording sites [radial, brachial (BA) and common carotid artery (CCA)], different waveform analysis algorithms (e.g., direct analysis of the CCA pulse waveform vs. peripheral waveform analysis using general transfer functions, N-point moving average filters, *etc.*), different calibration schemes [systolic-diastolic calibration vs. methods using BA diastolic and mean blood pressure (bMBP)]; the latter calculated using different equations vs. measured directly by oscillometry, and different equations to estimate bMBP. The invasive aortic (aoBP) and brachial pressure (bBP) (catheterization), and the non-invasive aoBP and bBP were simultaneously obtained in 34 subjects. Overall, non-invasive approaches yielded lower aoSBP and aoPP levels than those recorded invasively. Moreover, aoSBP and aoPP determinations based on CCA recordings, followed by BA recordings, were those that yielded values closest to those recorded invasively. However, most of the non-invasive approaches overestimated and underestimated aoSBP at low (i.e., 80 mmHg) and high (i.e., 180 mmHg) invasive aoSBP values, respectively. The higher the invasively measured aoPP, the higher the level of underestimation provided by the non-invasive methods. The authors highlighted that the recording method and site, the mathematical method/model used to quantify aoSBP and aoPP, and calibration of waveforms are essential when estimating aoBP. Therefore, the authors emphasized on the need for methodological transparency and consensus for the non-invasive aoBP assessment.

Menon et al. were interested to define a novel list index for vascular studies. The authors observed that the insights attained in clinical/population research from linking datasets on vascular function under different conditions, have not been fully realised in the basic sciences, thus frustrating advanced analytics and complex modelling. Therefore, to achieve comparable advances in basic sciences, the authors tried to define/measure integrated vascular function combining data across conditions, models, and groups. They described an approach to establish and validate a composite metric of vascular function by comparing parameters of vascular function in metabolic disease (using the obese Zucker rat model) to the same parameters in age-matched, “healthy” conditions, resulting in a common outcome measure which they termed the vascular health index (VHI). The VHI permits the integration of datasets, thus expanding sample size and promoting advanced modelling to gain insight into the development of peripheral and cerebral vascular dysfunction. Markers of vascular reactivity, vascular wall mechanics and microvascular network density are integrated in the VHI. The authors provided a detailed presentation of the development of the VHI and presented multiple measures to assess face, content, criterion and discriminant validity of the metric. The results demonstrated how the VHI captures multiple indices of dysfunction in the skeletal muscle and cerebral vasculature with metabolic disease and provided context for an integrated understanding of vascular function under challenged conditions.

The large variety of manuscripts submitted to Frontiers in Physiology—Vascular Physiology as part of this Research Topic highlighted the dynamism of our research field. The ever-evolving technologies as well as the mass of knowledge aggregated by the various research groups across the globe are pushing us to re-evaluate how we work, and giving the community the opportunity to share these advances in such a forum is of the utmost importance.

